# Advancing AI Competency in Graduate Medical Education: A Developmental Framework

**DOI:** 10.1016/j.focus.2026.100484

**Published:** 2026-02-04

**Authors:** Tauhid N. Mahmud, Yuri T. Jadotte, Dorothy Lane

**Affiliations:** 1 Department of Biomedical Informatics, Renaissance School of Medicine, Stony Brook University, Stony Brook, New York; 2 Division of Population Health and Community Medicine, Department of Family, Population and Preventive Medicine, Renaissance School of Medicine, Stony Brook University, Stony Brook, New York; 3 Division of Family and Preventive Medicine, Department of Family, Population and Preventive Medicine, Renaissance School of Medicine, Stony Brook University, Stony Brook, New York; 4 Division of Public Health, Department of Family, Population and Preventive Medicine, Renaissance School of Medicine, Stony Brook University, Stony Brook, New York; 5 JBI Population Health Evidence Consortium, School of Nursing, Rutgers University, Newark, New Jersey; 6 Office of Continuing Medical Education, Dean's Office, Renaissance School of Medicine, Stony Brook University, Stony Brook, New York

## INTRODUCTION

The integration of artificial intelligence (AI) into health care is no longer a scenario reserved for science fiction; it is rapidly becoming a critical component of clinical practice.^1^ A 2024 survey of 43 U.S. health systems found that 100% had implemented AI-based clinical documentation tools, whereas the Food and Drug Administration has authorized over 1,000 AI-enabled medical devices as of 2024—representing exponential growth from just 6 approvals in 2015 to 223 in 2023.^2^ As development and implementation accelerate, medical education confronts an urgent imperative to reimagine how to prepare current and future physicians for a landscape where AI tools increasingly influence healthcare policy, clinical decision making, documentation, and patient interaction. This influence extends beyond individual patient encounters, shaping resource allocation, public health strategies, and healthcare systems and processes, thus demanding a corresponding breadth of understanding from physicians.

Current residency curricula remain largely anchored in traditional clinical training models that inadequately address the AI transformation reshaping health care.^3^ Evidence suggests that AI education across undergraduate medical education, graduate medical education (GME), and continuing medical education, or continuing professional development is often characterized as a patchwork of initiatives, lacking uniformity, systematic integration, and standardized curricula.^3^ Many medical education systems have been slow to adapt, resulting in a paucity of AI-specific education, despite surveys indicating strong support among trainees and physicians for its inclusion.^3^ Most programs offer, at best, sporadic exposure to AI concepts through disconnected lectures or optional workshops. This approach risks creating a physician workforce that may use AI-powered tools but lacks the foundational knowledge and skills to evaluate them critically or engage meaningfully with their development. This growing divergence between the rapid pace of AI adoption in practice and the slow, inconsistent nature of AI education in GME signifies a potential decoupling of physician competency from the very tools reshaping their professional environment, undermining their capacity to critically assess, adapt to, and responsibly integrate these technologies into patient care. Nevertheless, residents and medical students' innate comfort with digital technologies, stemming from their generational exposure, may enable them to bridge the gap between clinical practice and AI innovation in ways that complement formal training.

Consider a typical internal medicine resident who encounters an AI-powered diagnostic support system suggesting an uncommon diagnosis for a patient with nonspecific symptoms. Without proper training, the resident lacks the framework to evaluate whether this recommendation stems from valid machine-learning techniques based on thousands of similar cases^4^ or from biased training data that overrepresent certain conditions or underrepresent specific patient populations.^5^ AI's diagnostic strength lies in its ability to analyze vast data sets, identifying complex patterns and correlations that might elude human analysis.^6^ However, it is well documented that if the training data are misrepresentative of population variability or lacking diversity in demographics, disease presentation, or psychosocial factors, AI algorithms are prone to reinforcing existing societal biases, potentially leading to misdiagnoses, inappropriate treatment recommendations, and the exacerbation of health disparities.^5^

Just as the Flexner Report reshaped undergraduate medical education^7^ and the Accreditation Council for Graduate Medical Education (ACGME) competencies and Milestones framework transformed residency training,^8^ medical education now faces a new challenge with AI. Addressing it requires not only technical instruction but also a comprehensive framework for developing critical AI competency. This competency should encompass understanding AI capabilities and limitations, recognizing potential biases and security infringements, evaluating appropriate use cases, identifying best practices, and maintaining human judgment in an increasingly automated environment.

## A PROPOSED FRAMEWORK TO GUIDE THE INTEGRATION OF AI INTO GRADUATE MEDICAL EDUCATION

The authors propose a framework that categorizes AI competency levels as Observers, Adopters, and Innovators, offering a structured pedagogic approach designed to integrate seamlessly with existing GME structures, particularly the ACGME Milestones. The framework acknowledges that not every physician will become an AI researcher but every physician must develop critical evaluation skills commensurate to their level of engagement with these technologies. As AI competency deepens from Observer to Adopter to Innovator, so too does the capacity to critique and contribute. These levels are mapped to the developmental progression outlined in the ACGME Milestones, providing a familiar structure for assessment and curriculum design that may facilitate widespread adoption (Table 1).

**Table 1:** Mapping Proposed AI Competency Levels to ACGME Milestone Framework

AI competency level	ACGME Milestone level	Description/focus	Example skills
Observer	Levels 1–2 (Novice–Beginner)	Foundational awareness of AI capabilities, limitations, and ethics	Identifies when AI tools are appropriately used; appropriately inquires about function and data sources; understands the role of AI in privacy and bias
Adopter	Levels 3–4 (Competent–Proficient)	Applies AI tools critically and appropriately	Evaluates AI performance; interprets outputs in context; identifies bias and integrates AI into workflow
Innovator	Level 5 (Expert)	Leads AI evaluation, development, and implementation	Designs QI projects; contributes to tool development; mentors peers; critically evaluates vendor claims

This table outlines a tiered framework for developing AI competency in graduate medical education, mapping progression from foundational awareness (Observer) to practical application (Adopter) and leadership in innovation (Innovator) to corresponding ACGME Milestone levels. Each tier includes representative skills and expected capabilities at each stage of training. These can also be incorporated into existing Systems-based Practice competency milestones.

ACGME, Accreditation Council for Graduate Medical Education; AI, artificial intelligence; QI, quality improvement.

The authors propose several key strategies to systematically optimize residency training for AI competency. First, AI competency training must be woven meaningfully into existing clinical education rather than siloed as a separate technological topic. For example, during a pediatric morning report on an asthma exacerbation, faculty could introduce an AI-powered monitoring tool that predicts deterioration hours before clinical signs appear. The discussion could then highlight both the benefits and limitations of such a system, such as how frequent false alarms may contribute to alarm fatigue and inappropriate interventions and how algorithm transparency, including knowing which data features drive the prediction, is essential for building clinician trust. Morning reports, noon lectures, and case conferences therefore provide natural opportunities to explore how AI tools may influence diagnosis, treatment planning, and workflow changes in real clinical scenarios.

Second, residency programs should create structured pathways for all trainees to progress from Observer to Adopter levels, with opportunities for those pursuing advanced scholarship to advance to Innovator roles. Imagine a surgical resident who begins by critically evaluating AI-powered imaging analysis tools (Observer), then implements a protocol for their use in preoperative planning (Adopter), and eventually collaborates with engineers to improve the tool's performance on diverse patient populations (Innovator). Supporting this progression includes actively involving residents in institutional AI initiatives, creating elective experiences focused on building or improving clinical AI workflows relevant across various specialties, and establishing pathways for diverse scholarly activities. These could include not only traditional research but also quality improvement projects, implementation science studies, and program evaluations for those interested in contributing to AI development and application. Recognizing diverse forms of scholarship beyond traditional research makes the Innovator role more accessible and acknowledges that valuable contributions to AI implementation can arise from methodologies already embedded within GME, such as quality improvement.

Third, critical evaluation skills specific to AI must be explicitly taught and assessed. Consider a scenario where emergency medicine residents are presented with an AI tool that claims to predict sepsis onset: they should be trained to ask about and evaluate the evidence on the tool's validation population, sensitivity and specificity, potential biases, and impact on clinical outcomes, just as they would critically evaluate a new medication or procedure. This requires moving beyond simple technical metrics such as accuracy, which often dominate evaluations, toward a more comprehensive assessment incorporating clinical impact, integration challenges, and economic sustainability.^9^ Residents should regularly practice analyzing AI outputs, identifying potential biases or errors, and determining appropriate clinical applications and limitations. This could include comparing AI recommendations with clinical guidelines, discussing whether the underlying validation data match the patient population, and considering how errors might influence care. It is important to highlight that trainees and faculty currently face significant challenges in conducting such evaluations because systematic assessment frameworks remain nascent and access to critical information about algorithmic design, training data, and validation processes is often restricted by proprietary limitations. Nevertheless, residency programs should teach trainees to ask critical questions even when complete answers are unavailable: What population was used for training and validation? How does the tool perform across different demographic groups? What is the evidence of clinical impact beyond technical accuracy?

Fourth, ethics and equity considerations must be central, not peripheral, to AI competency. For example, when discussing an AI dermatology diagnostic tool, faculty should highlight how algorithms trained predominantly on light-skinned patients may perform poorly on darker skin tones, potentially exacerbating existing healthcare disparities.^5^^,^^10^ This issue stems directly from biases in data acquisition and representation, where data sets often lack diversity or reflect historical inequities.^10^ Similarly, during a health department rotation, a preventive medicine resident analyzing county surveillance data for sexually transmitted infections might encounter an AI-powered risk stratification tool that flags certain ZIP codes as high risk for outbreak interventions. However, if this algorithm was trained on data that reflect historical patterns of testing accessibility, such as when certain communities had limited access to sexually transmitted infection screening, the tool may systematically under-identify transmission hotspots in underserved areas while oversurveilling communities that already receive more healthcare attention (i.e., historical bias).^11^ This could lead to misallocation of prevention resources and perpetuate the very disparities the public health intervention aims to address. The resident must critically evaluate whether the AI tool's recommendations align with principles of health equity and consider how historical healthcare access patterns may have shaped the training data. All discussions and implementations of AI tools should address questions of data representation, algorithmic bias, and known or potential impacts on healthcare disparities, recognizing that AI deployed without careful consideration risks amplifying inequities on a global scale.

As the technology produced by AI companies shapes healthcare delivery through AI-powered tools, the physician's role as critical evaluator and patient advocate becomes ever more crucial. Consider the hospitalist who must decide whether to implement an AI-powered readmission prediction tool: without critical AI competency, they cannot effectively evaluate vendor claims, assess potential benefits and harms, or advocate for necessary customizations to serve their specific patient population. Residents must be prepared not just to use these tools but to engage critically with their development, implementation, and regulation.

The proposed framework, integrated within the ACGME structure, recognizes this essential responsibility. By defining clear pathways for increasing AI competency, from basic awareness (Observer/Levels 1–2) to practical implementation (Adopter/Levels 3–4) to innovative development (Innovator/Level 5), it provides a roadmap for residency programs to ensure that all physicians develop appropriate levels of critical AI competency. Failure to prepare residents for this technological transformation risks creating a generation of physicians who either blindly trust algorithmic outputs or reflexively reject potentially beneficial tools. Neither approach serves patients, society, or the physician workforce itself. Instead, there is a need for physicians who can harness AI's capabilities while maintaining the critical thinking, ethical judgment, and human connection that define our profession.

Implementing this framework faces significant barriers that must be acknowledged. Most notably, many current faculties lack formal AI training, creating a critical need for faculty development. Potential solutions include establishing partnerships between medical education programs and clinical informatics or biomedical informatics departments to provide faculty training, creating train-the-trainer programs where early adopters mentor colleagues, and leveraging online educational resources and continuing medical education courses focused on AI in health care. In addition, residency programs could designate AI education champions, faculty members who receive protected time for curriculum development and peer education. Professional organizations such as specialty boards could support this effort by developing standardized faculty development curricula. In addition, the ACGME could support this effort by proposing the integration of AI competency into the milestones development process for every medical specialty, particularly within the Systems-based Practice competency domain where they would be a natural alignment with existing frameworks for evaluating healthcare systems, quality improvement, and patient safety. However, the authors recognize that the medical community may need intermediate steps before formal ACGME adoption. These interventions acknowledge that faculty development must occur in parallel with curricular changes, creating a learning environment where both trainees and faculty grow together in AI competency.

## CONCLUSIONS

The time to transform the GME approach to AI education is now, before practice patterns become entrenched and harder to modify. The proposed framework, explicitly aligning AI competency development with the established ACGME Milestones, offers a starting point for this essential evolution of GME. It provides a structured, integrated approach that recognizes the varying roles that physicians will play in an AI-enabled future while ensuring that all physicians develop the critical evaluation skills necessary to apply these powerful tools wisely, equitably, and ethically. Equipping the next generation of physicians with critical AI competency is not merely an educational update; it is fundamental to upholding the future integrity and effectiveness of the medical profession.

## CRediT authorship contribution statement

**Tauhid N. Mahmud:** Conceptualization, Writing – original draft, Writing – review & editing. **Yuri T. Jadotte:** Supervision, Conceptualization, Writing – review & editing. **Dorothy Lane:** Supervision, Writing – review & editing.
